# Severe Pyogenic Liver Abscess in a Diabetic Patient Presenting With Sepsis and Diabetic Ketoacidosis: A Case Report

**DOI:** 10.7759/cureus.92344

**Published:** 2025-09-15

**Authors:** Haytham Mohammed Alzinati, Ahmed Zaki, Ammar Mohamed Saleih Abdalah Basheir, Ahmed Fathi Mohamed Alsehily, Ahmed Elsayed Ibrahim Mattar, Abdulmalek Ayman Arbach, Shahad Yaser Mustafa, Abdullah Y Mustafa, Ahmed H Abdulhay

**Affiliations:** 1 General Surgery, Saudi German Hospital, Hail, SAU; 2 General Surgery, Ibn Sina University, Khartoum, SDN; 3 General Surgery, Sulaiman Al Rajhi University, Al Bukayriyah, SAU; 4 Surgery, Bolu Abant Izzet Baysal University, Bolu, TUR

**Keywords:** diabetes mellitus, diabetic ketoacidosis (dka), klebsiella pneumoniae, liver abscess drainage, severe sepsis

## Abstract

Pyogenic liver abscess (PLA) is a rare but potentially life-threatening condition, especially in immunocompromised and diabetic patients. Complications such as sepsis and diabetic ketoacidosis (DKA) increase morbidity and mortality among diabetic patients. We report the case of a 50-year-old male patient who presented with fever, confusion, and hypotension. Initial assessment revealed septic shock and DKA. Imaging confirmed a large liver abscess. Despite intensive medical therapy, laparoscopic drainage was required. Culture revealed *Klebsiella pneumoniae*. The patient required prolonged ICU care, insulin therapy, targeted antibiotics, and multidisciplinary management, after which he gradually improved. Early suspicion and timely intervention are lifesaving in complicated PLA cases. In patients with diabetes and sepsis, multidisciplinary care is crucial for optimizing outcomes.

## Introduction

Liver abscess is a serious infectious disease characterized by the presence of pus-filled cavities within the hepatic parenchyma. Among the causes of liver abscess, the pyogenic type accounts for the majority of cases, representing 60% of incidences [1-4]. Despite improvements in diagnostic imaging and antimicrobial therapy, pyogenic liver abscess (PLA) remains associated with significant morbidity and mortality, with case-fatality rates ranging between 3% and 30% [5].

PLA is caused by a spectrum of pathogens; however,* Klebsiella pneumoniae* is a leading cause, particularly in diabetic and immunocompromised patients [6-8]. Mortality rates for *Klebsiella pneumoniae* liver abscesses in diabetic patients vary, with some studies showing an overall in-hospital mortality of up to 25.0% [9]. Liver abscesses caused by *Klebsiella pneumoniae* are often associated with many chronic diseases, and studies have shown that diabetes mellitus is a common concomitant condition [10]. The organism has been linked to liver abscess and invasive syndromes characterized by metastatic spread to extrahepatic sites, including the lungs, central nervous system, and eyes [7,11].

The clinical presentation of PLA is highly variable, which contributes to delays in diagnosis [6,12-14]. Symptoms may include fever and chills, along with right upper quadrant abdominal pain [6]. Diagnostic modalities for PLA include imaging (ultrasonography and/or computed tomography) and needle aspiration [14]. Management of PLA typically requires a combination of broad-spectrum antimicrobial therapy and drainage. Percutaneous aspiration or catheter drainage, guided by ultrasound or computed tomography, is the preferred approach and has high success rates, reaching 87% to 92% [6,12-15]. However, in cases with multiloculated abscesses, poor organization of the collection, or failed percutaneous attempts, surgical drainage remains the definitive treatment [6,12-15].

Here, we present the case of a 50-year-old male patient who developed a severe PLA complicated by sepsis and diabetic ketoacidosis (DKA). This case emphasizes the diagnostic and therapeutic challenges of managing this condition in a diabetic patient and highlights the importance of timely imaging, prompt initiation of antimicrobial therapy, and the role of surgical intervention when less invasive measures fail.

## Case presentation

A 50-year-old male patient presented on February 26, 2025, with fever, confusion, epigastric pain, and hypotension. On arrival, his vital signs were: blood pressure 80/60 mmHg, heart rate 111 beats per minute, temperature 39.9°C, and oxygen saturation 93%. 

Initial laboratory tests revealed metabolic acidosis and a random blood sugar of 429 mg/dL. The patient also had elevated C-reactive protein at 44.77 mg/dL and procalcitonin at 72.83 ng/mL, as well as elevated alanine aminotransferase at 171 U/L and aspartate aminotransferase at 154 U/L (Table 1). Hemoglobin A1c was 10.6%, confirming poorly controlled diabetes mellitus. The patient was admitted to the ICU and treated with intravenous fluids, insulin infusion according to the DKA protocol, and empirical antibiotics.

**Table 1:** Laboratory findings MCV: Mean corpuscular volume; MCH: Mean corpuscular hemoglobin; MCHC: Mean corpuscular hemoglobin concentration; RDW: Red blood cell distribution width; CRP: C-reactive protein

Parameter	Results	Normal range
Random blood glucose (mg/dL)	429	70-150
CRP (mg/dL)	44.77	0-5
Lactate (mg/dL)	25.5	4.5-19.8
Procalcitonin (ng/mL)	72.83	0-0.07
Alanine aminotransferase (U/L)	171	0-55
Aspartate aminotransferase (U/L)	154	5-34
White blood cells (× 10³/µL)	12.2	4-11
Red blood cells (× 10⁶/µL)	4.23	4.7-6
Hemoglobin (g/dL)	12.8	13.5-18
Hematocrit (%)	38.1	42-52
MCV (fL)	90.2	83-99
MCH (pg)	30.2	27-32
MCHC (g/dL)	33.5	31.5-34.5
RDW (%)	11.6	13.5-14.5
Platelet count (× 10³/µL)	115	150-400
Neutrophils (%)	68.1	50-62
Lymphocytes (%)	15.3	25-40
Monocytes (%)	16.1	3-7
Eosinophils (%)	0.06	0-1
Basophils (%)	0.44	0-1

**Figure 1:**
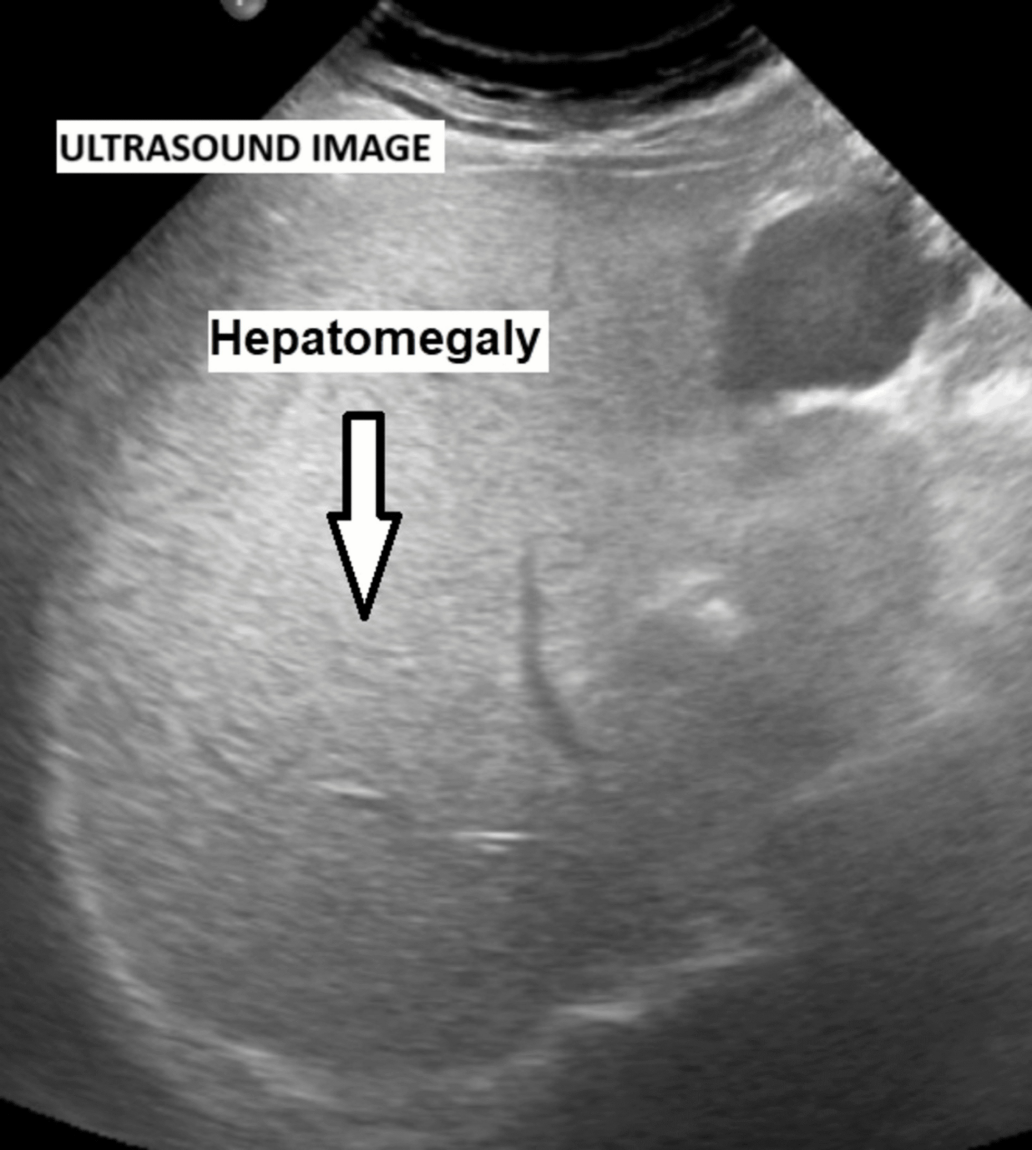
Abdominal ultrasound scan showing hepatomegaly

During admission, the patient’s vital signs improved, with blood pressure 110/60 mmHg, heart rate 80 beats per minute, oxygen saturation 96%, and random blood sugar of 190 mg/dL. However, the patient continued to experience fever, epigastric pain, and high inflammatory markers. An abdominal ultrasound performed on the second day of admission revealed hepatomegaly with a homogeneous echo pattern but was unsuccessful in identifying a focal lesion (Figure 1). While awaiting blood culture results, he required ongoing broad-spectrum antibiotic therapy (initially with ceftazidime, which was later escalated to piperacillin-tazobactam).

**Figure 2:**
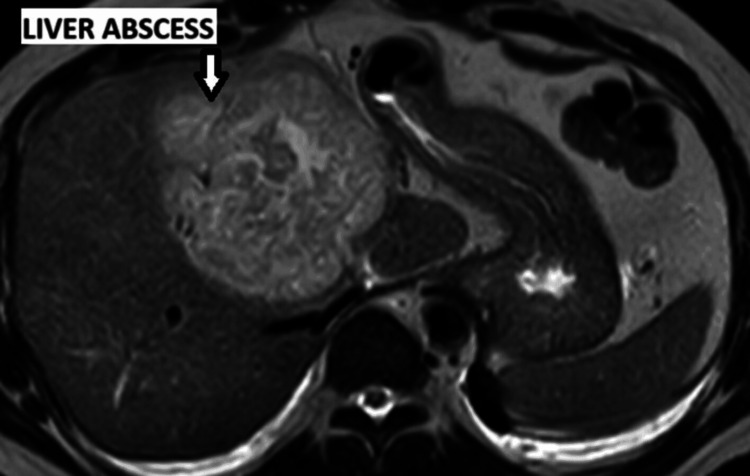
MRI of the abdomen showing a large liver abscess

Between March 1 and March 3, the patient continued to have recurrent episodes of fever and persistently elevated inflammatory markers. Another imaging study with MRI demonstrated a large and well-defined hepatic focal lesion measuring 9.0 × 8.3 cm. The lesion involved the right liver lobe (segment V) and extended to the medial segment of the left liver lobe (segment IV), consistent with a liver abscess (Figure 2). Interventional radiology was consulted, but the collection appeared phlegmonous and poorly organized, making it unsuitable for percutaneous drainage with a pigtail catheter. Consequently, the patient was scheduled for laparoscopic surgical drainage.

**Figure 3:**
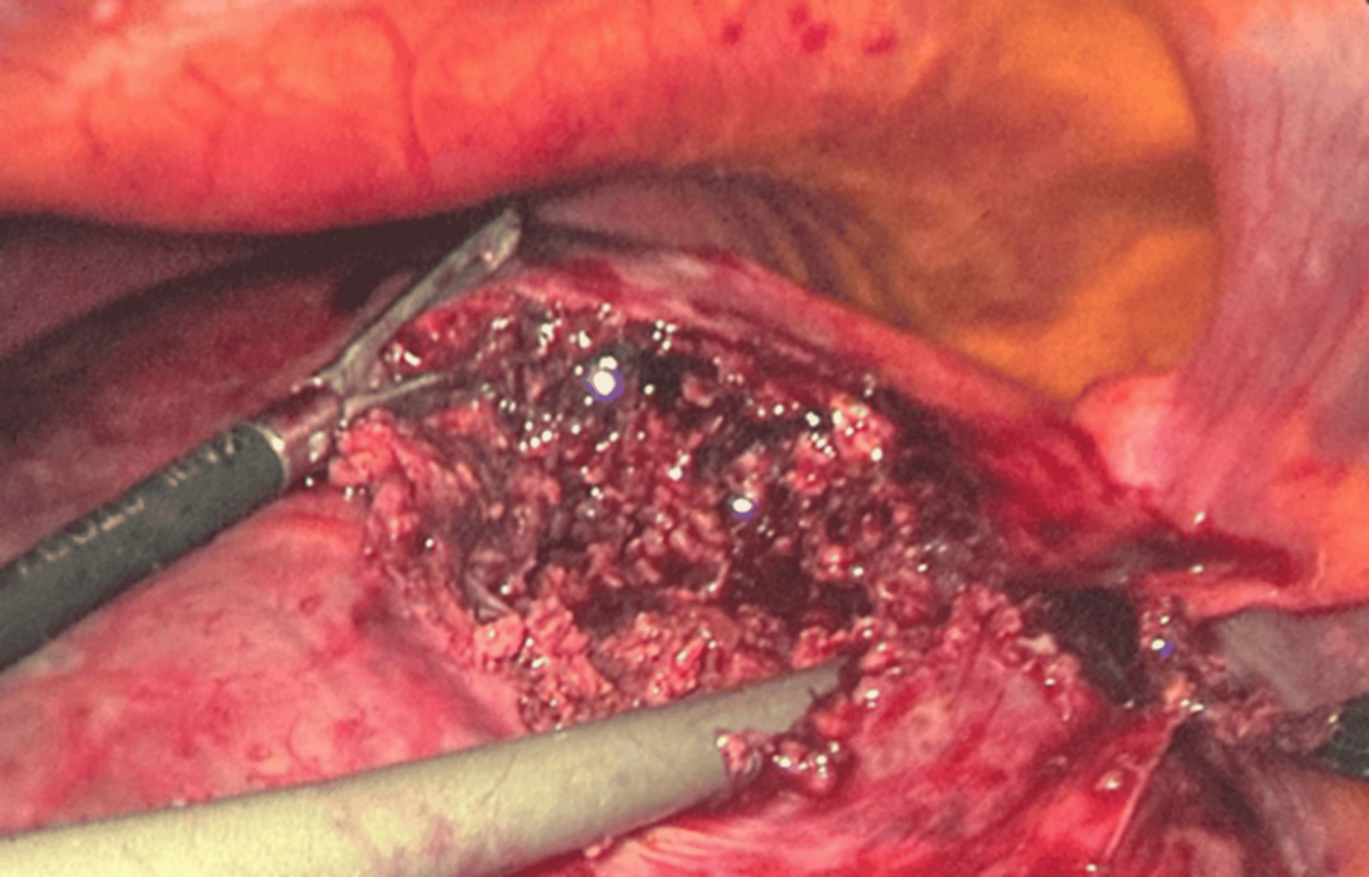
Intraoperative image demonstrating debridement and drainage of a pyogenic liver abscess with drain insertion

On March 5, 2025, the patient underwent laparoscopic exploration under general anesthesia for management of the liver abscess. The pneumoperitoneum was established using a Veress needle (Ethicon, Johnson & Johnson, USA) inserted at Palmer’s point. An 11 mm infraumbilical port (Applied Medical, USA) was introduced for the laparoscopic camera. Upon entry into the peritoneal cavity, a large bulge was noted on the superior surface of the liver. A transverse incision was made over the bulge and deepened until copious pus was encountered. The cavity was widened, followed by debridement and irrigation of the liver parenchyma. A 16 Fr Jackson-Pratt drain (Cardinal Health, USA) was inserted into the abscess cavity (Figure 3). The incision site was oversewn with a single interrupted vicryl 2/0 suture to secure the drain. Irrigation and suction of the peritoneal cavity were performed, and all ports were removed under direct vision. Finally, skin closure and dressing were completed.

After the operation, the patient was admitted to the ICU due to hypotension and tachycardia. The drain output was approximately 100 mL of turbid pus containing necrotic debris. The culture grew *Klebsiella pneumoniae*, which was sensitive to tigecycline and piperacillin-tazobactam. Subsequently, he was treated with targeted antibiotics.

The patient showed gradual clinical improvement, and the drain output decreased. Glycemic control was optimized, and liver enzyme derangements began to improve. The white blood cell count fluctuated between 14-19 × 10^9^/L in the early course before gradually declining to around 11 × 10^9^/L. C-reactive protein remained elevated between 18 mg/dL and 31 mg/dL before showing improvement. Liver enzymes, which initially peaked at 100 U/L and 145 U/L for alanine aminotransferase and aspartate aminotransferase, respectively, later normalized. Renal function remained stable throughout hospitalization.

By the second week of March, the patient was conscious, oriented, and vitally stable. He tolerated an oral diet, continued on intravenous antibiotics, and had decreasing drain output. The drain was removed, and the patient was discharged after antibiotic therapy was completed and sepsis was fully resolved.

## Discussion

PLA is a significant health concern, with increasing incidence worldwide and a mortality risk, with case-fatality rates ranging between 3% and 30% [5]. This case highlights the challenges of diagnosing and managing PLA in a diabetic patient presenting with sepsis and DKA. *Klebsiella pneumoniae* is increasingly recognized as the predominant pathogen in diabetic patients and is associated with severe infections, septicemia, and potential metastatic complications [7].

Diabetes mellitus is one of the strongest predisposing factors for PLA. Poor glycemic control weakens host immunity by impairing neutrophil activity, phagocytosis, macrophage function, and antibody/complement responses, thereby increasing susceptibility to infections. Hyperglycemia also enhances bacterial virulence, as seen in *Klebsiella pneumoniae*, by upregulating virulence genes and promoting capsular polysaccharide production, which improves resistance to immune defenses [16-19]. Therefore, PLA may present more aggressively in such patients and is more frequently complicated by bacteremia, sepsis, or DKA, which further worsens outcomes [7].

The clinical presentation of PLA is highly variable. While fever, chills, and right upper quadrant pain are classical findings, many patients present with nonspecific symptoms or with extrahepatic features such as septic shock or confusion [14]. This nonspecificity could lead to delays in diagnosis. Imaging with ultrasound or computed tomography is the cornerstone for diagnosis, as laboratory findings alone are neither sensitive nor specific [8].

Management of PLA typically requires a combination of broad-spectrum antimicrobial therapy and drainage. Percutaneous aspiration or catheter drainage, guided by ultrasound or computed tomography, is the preferred approach and has high success rates, ranging from 87% to 92% [4,15]. However, in cases with multiloculated abscesses, poor organization of the collection, or failed percutaneous attempts, surgical drainage remains the definitive treatment [6,7]. Even though a nonoperative interventional radiology approach has become the first therapeutic choice for PLA, surgical treatment is still necessary in some cases.

In this case, the percutaneous catheter drainage was not feasible due to the nonorganized nature of the abscess. Therefore, laparoscopic surgical drainage became the definitive management strategy. This approach is supported by international guidelines such as those from the European Association for the Study of the Liver and the Infectious Diseases Society of America, which recommend surgical intervention in cases where percutaneous drainage fails or is not technically possible [6,13,14].

The coexistence of DKA and sepsis in this patient compounded disease severity. DKA requires prompt recognition and management with fluid resuscitation, electrolyte replacement, and insulin infusion, while concurrent sepsis mandates early broad-spectrum antimicrobial therapy and source control. The simultaneous management of these critical conditions emphasizes the importance of multidisciplinary collaboration between intensive care physicians, infectious disease specialists, radiologists, and surgeons.

## Conclusions

PLA is a serious infection associated with significant morbidity and mortality, particularly in diabetic patients. Despite the high risk of poor outcomes, early diagnosis and treatment with antibiotics and drainage significantly improve the prognosis. This case highlights the importance of early recognition and diagnosis, timely initiation of antibiotics, and the need for surgical drainage when percutaneous methods are unsuccessful. Multidisciplinary management, including strict glycemic control, infection control, and intensive care support, is essential for optimizing patient outcomes.
